# Bibliography

**Published:** 1900-05-15

**Authors:** 


### BIBLIOGRAPHY.

The fifth edition of the "Practice Builder," a treatise on the conduct of a dental practice, has just been put into our hands. It is a large octavo of nearly 700 pages, printed on good paper and well done from the printer's and binder's point of view. Its editor, Dr. Charles R. Hambly, suggests to the reader that to get the most out of the book it will be necessary to read and study it carefully from cover to cover.

We have followed this advice, and after passing the six different introductory statements and the index, we take up Chapter I on the subject of Dental Education, and find it full of meat of a very interesting character and containing a well-arranged statement of educational affairs, statistically, historically and philosophically. This chapter contains statements of facts upon which volumes have been or may be written.

The chapter on Dental Legislation is not so complete a statement of the function of legislation as should or could be made.

The chapter on selecting a place in which to build a practice is a good one and will pay a young man for the cost of the book, provided he will act in harmony with the valuable ideas given and suggested. We do not think the author puts enough value on the practice of the new man, either associating himself with an old practitioner for a time or by purchasing outright a practice, both of which are invaluable methods of getting into practice.

The outfit and furnishing of the office are each carefully commented upon, and many valuable suggestions given.

A long and valuable chapter on the elements of success contains a very conservative statement, and some of the causes for failure are clearly stated, and we think the author wisely attributes to *ignorance* the chief cause of failure.

The personality of the dentist is well-discussed and his character, habits and personal appearance each are given a show in making up a fully rounded professional man.

Considerable space is given to the subject of advertising as a means specially of introducing a new practitioner to the public. A large variety and number of printed notices are given as samples. Some of them we should not like to vouch for as entirely professional or in strict accord with the National Code of Ethics. In the chapter devoted specially to advertisement writing some clever suggestions are made as to advertisement writing, etc. In a foot note the author explains that he does not commend this kind of advertising, but he has taken the trouble to collect many specimens from all parts of the world, and from this data he draws his conclusions as the proper method of writing advertisements which will attract people and bring business to the office. The ethics of the question he leaves out altogether, because, as he says, he is writing a book for all classes and times. It is possible the book would be just as useful had this chapter been left out. The article on stationary, cards, etc., contains many useful and wise suggestions.

The question of compensation for services rendered is very well handled, and some good suggestions are made for getting rid of undesirable or discount patients.

The chapter on the use and abuse of credit is perhaps one of the most important in the book, and should be well considered by every young man who is forming business habits, and it would be a very wise move for many practitioners to buy this book and study carefully the suggestions made. We are sure they would be put thinking, and to some good purpose.

There is so much that is worth commending in the book that we have not space to call attention even to, that we can only suggest that while the book contains much to which we could take exception, it also contains much of great value, and is well worth the time necessary for its reading.

The Transactions of the National Dental Association for 1899 are at hand. In comparison with former volumes it is rather an extensive enlargement. It, however, also contains the proceedings of the Southern Branch, which takes up 231 of the 700 pages which make up the book. It is, as usual, well printed and bound, and the committee and the S. S. White Dental Manufacturing Company deserve praise for the care exercised in putting the records in such admirable form for reference and preservation. While most of the matter has already appeared in the *Cosmos* and other journals, it makes good reading, and will be invaluable in the future as a reference volume. This volume will serve as a record made by the administration under President H. J. Burkhart. There probably has never been a meeting of our National organizations having a larger attendance, nor one to which so many valuable papers were contributed.

